# Associations Between Lower‐Body Fast Maximal Dynamic and Reactive Strength Tests With Acceleration Performance in Semi‐Professional Male Soccer Players

**DOI:** 10.1002/ejsc.70056

**Published:** 2025-11-28

**Authors:** Jorge Rubio‐Lopez, Víctor Paredes‐Hernández, Javier Sánchez‐Sánchez, Carlos Balsalobre‐Fernández

**Affiliations:** ^1^ Department of Sport Sciences, Faculty of Medicine, Health and Sports Universidad Europea de Madrid Villaviciosa de Odon Madrid Spain; ^2^ Applied Biomechanics and Sports Technology Research Group Department of Physical Education Sport and Human Movement Autonomous University of Madrid Madrid Spain

**Keywords:** countermovement jump, modified reactive strength index, strength capacities, worst‐case scenario

## Abstract

The purpose of this study was to investigate the associations of fast maximal dynamic strength (MDS) and reactive strength capacity with physical match performance. Seventeen male semi‐professional soccer players (age = 20.2 ± 1.3 years) performed the countermovement jump (CMJ), drop jump and a modified version of the single‐leg triple crossover hop (CO_av_) tests during the season to measure their fast MDS and reactive strength capacities. Strength capacities were compared with the physical match performance measured through Global Positioning System (GPS) in official matches ± 14 days after the test day. The significance level was set at *p* < 0.05. CMJ significantly predicted the relative number of high‐intensity accelerations >4 m/s^−2^ (ACC_>4_) (*R* ^2^ = 42.9%; *p* < 0.01) and maximum acceleration (ACC_max_) (*R* ^2^ = 30.6%; *p* < 0.05). The transformed modified reactive strength index (log_10_ (mRSI)) also significantly predicted ACC_>4_ (*R* ^2^ = 37.1%; *p* < 0.01) and ACC_max_ (*R* ^2^ = 26.3%; *p* < 0.05). CO_av_ significantly predicted the worst‐case scenario for player load (WCS_PL_) (*R* ^2^ = 27.0%; *p* < 0.05). The present results suggest that different lower‐body strength capacities are associated with acceleration match performance.

## Highlights

- In soccer, countermovement jump (CMJ) and drop jump (DJ) performances are associated with maximal acceleration (ACC_max_) and high‐intensity accelerations (ACC_>4_), with CMJ showing stronger associations.
- The single‐leg triple crossover hop test (CO) can identify players who experience lower external acceleration‐derived loads during worst‐case match scenarios.
- Overall, jump‐based assessments (CMJ, DJ, CO) represent practical tests to infer acceleration‐related physical performance.

## 1 Introduction

Soccer is a dynamic sport characterised by frequent changes in intensity and a wide range of physical, technical and tactical factors that influence team performance (Bangsbo et al. 2006). Over the past decade, research has shown a significant increase in the physical demands of elite‐level matches, particularly in high‐intensity actions (Allen et al. 2024). Match analysis traditionally measures distances covered in speed zones, with high‐speed running and sprinting as key metrics for distinguishing players across competitive levels (Di Salvo et al. 2010, 2009). However, this approach often overlooks short, intense movements such as accelerations and decelerations. Although these actions fall below the high‐velocity threshold, they are more physically demanding than running at constant speed (Gaudino et al. 2013; Varley et al. 2012; Osgnach et al. 2024). These actions are key to performance and are associated with neuromuscular fatigue (Newans et al. 2019; Harper et al. 2019).

Global Positioning System (GPS) now allows for detailed tracking of accelerations, decelerations, velocity, distance and worst‐case scenarios (Torres‐Ronda et al. 2022). These actions are key components of high‐intensity external workload in elite soccer (Ravé et al. 2020) and failing to monitor these actions could lead to an underestimation of the physiological strain imposed by the sport. Accelerations and decelerations are essential parts of the external load, yet they impose different internal demands on the athlete. Accelerations, driven by concentric muscle contractions, incur a higher metabolic cost, whereas decelerations generate greater mechanical load due to the high‐impact forces involved (Hader et al. 2016; Dalen et al. 2016). Elite athletes often sustain these actions at higher frequency and intensity than lower‐level players (Draganidis et al. 2015; Johnston et al. 2015).

Although some studies have linked strength capacity to the ability to perform high‐intensity actions (Altmann et al. 2018; Rebelo et al. 2016), the evidence remains inconclusive, with other studies failing to establish strong correlations (Silva et al. 2013). Additionally, although player load (PL) is a useful indicator of external load, its relationship to strength remains underexplored (Oliva‐Lozano et al. 2023). Developing neuromuscular strength is a fundamental goal in physical training for athletes, as it supports key soccer movements, such as jumping, sprinting and changes in direction (Haff and Stone 2015). Furthermore, increased strength levels help athletes resist fatigue and reduce the risk of injury (Suchomel et al. 2016; Suchomel et al. 2018). Although some studies have explored the relationship between strength capacities and soccer performance, a gap remains in linking specific strength traits to match actions such as accelerations and decelerations. This gap may be due to the complexity of match contexts, variability in tracking technologies and performance metrics and a lack of standardised strength assessments that closely replicate game‐specific demands.

Although strength capacities can be distinguished into different manifestations, it has been argued that soccer strength tests should replicate the movements encountered during match play (James et al. 2023). Thus, tests of fast maximal dynamic strength (MDS) and reactive strength may better capture high‐intensity soccer actions. Additionally, strength can be classified by force direction—horizontal versus vertical (Ball and Zanetti 2012; Kotsifaki et al. 2021). Focusing on key strength capacities and force direction can help optimise training and enhance soccer performance.

For the reasons outlined, this study primarily aims to examine the relationship between athletes' fast MDS, reactive strength capacities and soccer performance, particularly in accelerations. A secondary aim is to identify which strength capacities explain variance in match acceleration performance. We hypothesise that fast MDS and reactive strength capacities are linked to high‐intensity match actions and account for their variance.

## 2 Methods

### 2.1 Subjects

**FIGURE 1:**
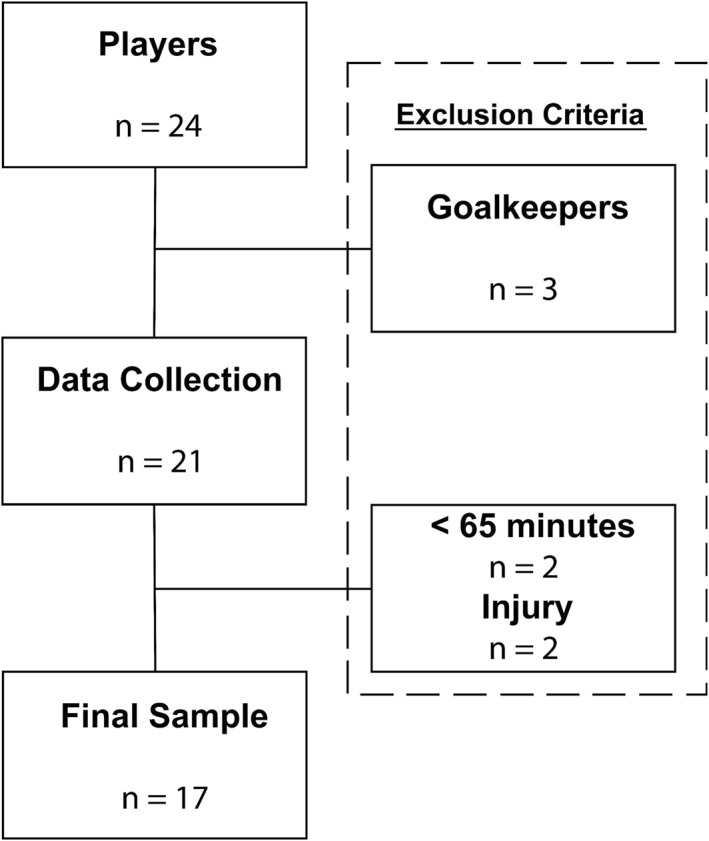
Study flow chart. The team consisted of 24 players: goalkeepers, players with insufficient playing time and players with a recent history of injury were removed from the analyses. Injuries represent one hamstring injury and one hip flexor injury.

The sample consisted of 17 male football players from a Spanish semi‐professional team (mean ± SD): age = 20.2 ± 1.3 years; height = 182.5 ± 6.2 cm; body mass = 75.2 ± 5.7. The subjects had been involved in soccer training 4–5 times a week for the past 2 years and performed gym‐based lower‐body strength training at least once a week. All nutrition and supplementation were monitored by the club's nutritionist, whereas any medication intake was supervised by the team's medical staff. The exclusion criteria were as follows: participants with lower‐limb injuries within the past 2 months, goalkeepers and players who did not play at least 60 min in an official match. From the 24 players in the squad, 17 were analysed (Figure 1).

This sample size aligns with prior studies on strength and physical match performance in soccer (Rebelo et al. 2016; Rago et al. 2018; Pedersen et al. 2022). Additionally, a previous power analysis (G*Power, *α* = 0.05, power = 0.80) indicated that detecting a strong correlation (*r* = 0.5) would require a minimum of 23 participants. Although our sample size of 17 falls below the threshold estimated by the power analysis, it remains sufficient to detect large effects, which can be identified with smaller sample sizes due to their stronger statistical signal. Participants received detailed information about the study's objectives and procedures, which were delivered verbally and in writing. Participants were free to withdraw their participation at any point without penalty. Furthermore, this study was conducted in accordance with the Declaration of Helsinki and was approved by the ethics committee of the University of Castilla–La Mancha [Spain] (CAU‐732752‐Z1C6).

### 2.2 Procedures

**FIGURE 2:**
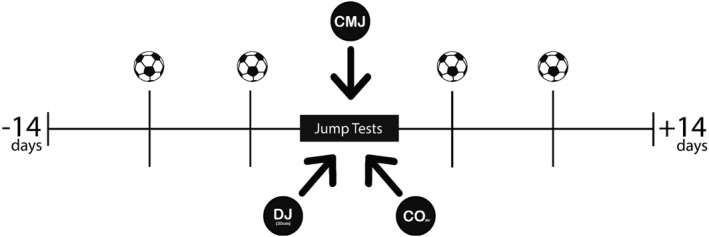
Visual representation of the data collection timeline.

All strength tests were performed during one visit to the team's gym facility before the field training session. Participants completed a countermovement jump (CMJ), a 30‐cm drop jump (DJ) and a modified single‐leg triple crossover hop test (CO). Match physical performance was analysed from all official matches played ± 14 days of the strength testing session (Figure 2).

#### 2.2.1 Strength Tests

Participants completed a 10‐min RAMP (raise and activate, mobilise and potentiate) warm‐up prior to testing. All tests were conducted mid‐season prior to the winter break, in the morning (∼2 h before field training, between 9:00 and 11:00 a.m.), in a controlled gym environment. Each test was supervised by at least two experienced fitness coaches, with over 5 years of professional experience, giving the same feedback to all participants. Participants performed two familiarisation jumps to minimise learning effects and enhance reliability.

#### 2.2.2 Fast Maximum Dynamic Strength (Vertical)—Countermovement Jump

**FIGURE 3:**
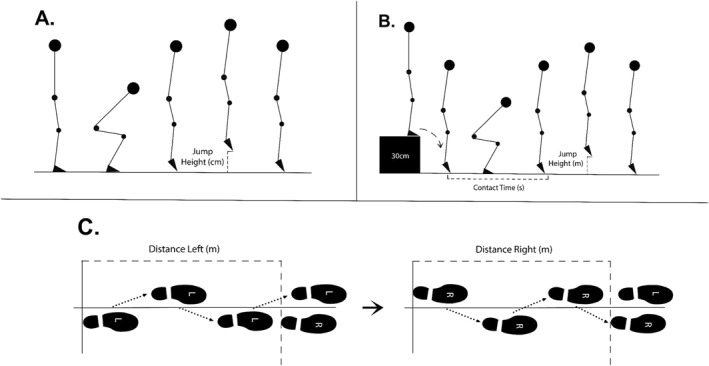
Visual representation of the (A) countermovement jump, (B) 30‐cm drop jump and (C) modified crossover tests.

CMJ height was used to assess fast MDS in the vertical force production plane (James et al. 2023). CMJ performance was measured using an IR OptoJump system (Microgate Co., Version 1.13, Bolzano, Italy). The participants stood between the infrared sensors with hands on hips. They were instructed to squat to approximately 90° at the knee and hips, then jump vertically as high as possible. For a visual representation, see Figure 3A. Three trials were completed, with 1 minute of rest between trials. The highest jump was recorded for analysis.

#### 2.2.3 Reactive Strength (Vertical)—Drop Jump

The DJ contact time and jump height were measured to quantify the reactive strength capacity of athletes in the vertical force production plane (James et al. 2023). A 30‐cm drop height was used, as it is commonly utilised to assess reactive strength in soccer players, balancing stretch‐shortening cycle engagement with safety (Flanagan and Comyns 2008). Participants stood on the box, stepped off and landed within the OptoJump system with both feet simultaneously. They were instructed to perform a maximal vertical jump with minimal contact time, keeping their hands on their hips throughout. For a visual representation, see Figure 3B. Three trials were completed, with 1 minute of rest between attempts. The ratio between jump height (m) to contact time (s) was used to calculate the modified reactive strength index (mRSI). The highest mRSI was used for analysis.

#### 2.2.4 Fast Maximum Dynamic Strength (Horizontal)—Single‐Leg Triple Crossover Hop Test

The CO was used to assess fast MDS in the horizontal force production plane (Kotsifaki et al. 2021). This test has shown good reliability and validity for assessing horizontal power and functional performance in football, especially for sprinting and change‐of‐direction ability (Lockie et al. 2012). Participants stood with one heel at the starting line and, using the same leg, performed three consecutive hops, each forward and diagonally across the centre line, and landed on both legs, one on each side of the line. Participants were instructed to stabilise and hold the landing for 2 seconds after. Three trials were performed on each leg, with a one‐minute rest period between trials. The landing distance (heel closest to the start line) was measured to the nearest centimetre. The best trial from each leg was used to calculate the average single‐leg triple crossover hop test (CO_av_). For a visual representation, see Figure 3C.

#### 2.2.5 Match Physical Performance

Data were collected from in‐season matches from a semi‐professional competition. The fixtures were scheduled on weekends and took place on natural grass fields. Before each match, participants completed a 20‐min warm‐up including physical, passing, possession and finishing drills. During matches, players wore GPS devices (Catapult Sports, Vector S7, Melbourne, Australia), with a sampling frequency of 10 Hz. Devices were calibrated in accordance with the manufacturer's instructions. Signal quality, horizontal dilution of precision and satellite count were checked post‐session to confirm adherence to manufacturer standards. All matches occurred on natural grass under sunny conditions, supporting GPS data reliability. Accelerations were defined as efforts exceeding 5 km/h lasting ≥ 0.9 s, with the best‐fit line (*r* >0.8) to ensure data quality. Data with poor reliability based on established signal quality and positional metrics were excluded from this study. GPS parameters (Table 1) were obtained using OpenField software (version 3.9.1, Catapult Sports Pty Ltd, Melbourne, Australia).

**TABLE 1:** GPS parameters to determine physical match performance.

Abbreviation	Parameter	Definition	Units
ACC_max_	Maximum acceleration	The maximum capacity of an athlete to accelerate for at least 1 s.	m/s^2^
DEC_max_	Maximum deceleration	The maximum capacity of an athlete to decelerate for at least 1 s.	m/s^2^
*V*_max_	Maximum velocity	Maximum velocity sustained by an athlete for at least half a second.	km/h
ACC_>4_	Relative number of high‐intensity accelerations	The average number of acceleration efforts above 4 m/s^2^ per minute of the match.	Count/min
DEC_>−4_	Relative number of high‐intensity decelerations	The average number of deceleration efforts above −4 m/s^2^ per minute of the match.	Count/min
PL/min	Player load per minute	The average PL per minute of the session. PL is ‘the sum of the accelerations across all axes of the internal triaxial accelerometer during movement. It considers the instantaneous rate of change of acceleration and divides it by a scaling factor (Bredt et al. 2020)’.	AU/min
m/min	Meterage per minute	The average running distance covered per minute of the session.	m/min
HSR_>21_	High‐speed running distance	The average distance covered running above 21 km/h per minute of the match.	m/min
WCS_dist_	Worst‐case scenario for distance	The greatest amount of distance covered in the match for a 3‐min rolling average interval.	m
WCS_PL_	Worst‐case scenario for player load	The greatest amount of PL accumulated in the match for a 3‐min rolling average interval.	AU

Abbreviations: AU = arbitrary units, PL = player load.

### 2.3 Statistical Analyses

The Shapiro–Wilk test was used to assess the normality of strength test results and GPS‐derived match performance data, appropriate for small samples (*n* < 50) (Mishra et al. 2019). mRSI data were log_10_‐transformed due to non‐normality (*p* < 0.05) to reduce skewness for robust analysis. After log_10_ transformation, log_10_ (mRSI) met normality assumptions (*p* >0.05). All other variables were normally distributed (*p* >0.05), supporting the use of parametric tests. Pearson correlations assessed relationships between strength tests and match performance metrics. Correlation coefficients were trivial (< 0.1), small (0.1–0.3), moderate (0.3–0.5), strong (0.5–0.7), very strong (0.7–0.9) and nearly perfect (>0.9). Linear regression was used to examine the predictive relationship between strength tests (independent) and match performance (dependent). Cohen's *f* ^2^ was calculated to assess the effect size, with thresholds for interpretation as follows: small (*f* ^2^ = 0.02), medium (*f* ^2^ = 0.15) and large (*f* ^2^ = 0.35). Statistical significance was set at *p <* 0.05. All statistical analyses were performed using RStudio (version 2024.04.2, Posit software, PBC).

## 3 Results

Mean, standard deviations (SD) and interquartile ranges of the strength tests and GPS‐derived physical match performance metrics are presented in Table 2.

**TABLE 2:** Strength test results for the soccer players and physical match performance on official matches (*N* = 17).

Strength test	Mean ± SD	Interquartile ranges _25%–75%_
CMJ (cm)	41.4 ± 3.8	38.5/43.0
mRSI (m/s)	1.40 ± 0.30	1.16/1.61
Log_10_ (mRSI)	0.1371 ± 0.0898	0.0626/0.2029
CO_av_ (m)	7.02 ± 0.36	6.79/7.28

Physical performance	Mean ± SD	Interquartile ranges _25%–75%_
ACC_max_ (m/s^2^)	4.76 ± 0.28	4.54/5.07
DEC_max_ (m/s^2^)	−5.98 ± 0.75	−5.62/−6.43
*V*_max_ (km/h)	31.9 ± 1.2	31.2/32.7
ACC_>4_ (count/min)	0.050 ± 0.029	0.031/0.073
DEC_>−4_ (count/min)	0.169 ± 0.070	0.127/0.217
PL/min (AU/min)	11.54 ± 2.18	10.40/13.22
m/min (m/min)	113.50 ± 11.72	106.00/121.15
HSR_>21_ (m/min)	6.40 ± 1.88	5.16/7.91
WCS_dist_ (m)	489.58 ± 34.66	467.81/514.87
WCS_PL_ (AU)	53.90 ± 6.78	48.43/61.50

Abbreviations: ACC_>4_ = accelerations above 4 m/s^2^, ACC_max_ = maximum acceleration, CMJ = countermovement jump, CO_av_ = average single‐leg triple crossover hop test, DEC_>−4_ = decelerations above −4 m/s^2^, DEC_max_ = maximum deceleration, HSR_>21_ = high‐speed running distance above 21 km/h, m/min = meterage per minute, mRSI = modified reactive strength index, PL/min = player load per minute, *V*_max_ = maximum velocity, WCS_dist_ = worst‐case scenario for distance, WCS_PL_ = worst‐case scenario for player load.

### 3.1 Correlations

The calculated correlations between match physical performance and strength capacities are presented in Table 3.

**TABLE 3:** Correlation between physical match performance and strength tests.

Physical performance	Strength test	Correlation (95% CI)
ACC_max_ (m/s^2^)	CMJ	*r* = 0.553* (0.098/0.817)
	Log_10_ (mRSI)	*r* = 0.513* (0.043/0.797)
	CO_av_	*r* = 0.109 (−0.395/0.558)
DEC_max_ (m/s^2^)	CMJ	*r* = 0.016 (−0.493/0.468)
	Log_10_ (mRSI)	*r* = −0.311 (−0.689/0.199)
	CO_av_	*r* = 0.009 (−0.474/0.487)
*V*_max_ (km/h)	CMJ	*r* = 0.325 (−0.184/0.697)
	Log_10_ (mRSI)	*r* = 0.288 (−0.223/0.675)
	CO_av_	*r* = −0.048 (−0.516/0.444)
ACC_>4_ (count/min)	CMJ	*r* = 0.655** (0.254/0.864)
	Log_10_ (mRSI)	*r* = 0.609** (0.182/0.843)
	CO_av_	*r* = 0.149 (−0.361/0.584)
DEC_>−4_ (count/min)	CMJ	*r* = 0.450 (−0.039/0.765)
	Log_10_ (mRSI)	*r* = 0.347 (−0.161/0.709)
	CO_av_	*r* = 0.247 (−0.272/0.646)
PL/min (AU/min)	CMJ	*r* = 0.023 (−0.463/0.498)
	Log_10_ (mRSI)	*r* = 0.115 (−0.387/0.565)
	CO_av_	*r* = −0.128 (−0.571/0.379)
m/min (m/min)	CMJ	*r* = 0.158 (−0.349/0.594)
	Log_10_ (mRSI)	*r* = 0.276 (−0.236/0.668)
	CO_av_	*r* = 0.204 (−0.312/0.620)
HSR_>21_ (m/min)	CMJ	*r* = 0.140 (−0.366/0.581)
	Log_10_ (mRSI)	*r* = 0.299 (−0.212/0.682)
	CO_av_	*r* = −0.130 (−0.572/0.377)
WCS_dist_ (m)	CMJ	*r* = 0.342 (−0.707/0.166)
	Log_10_ (mRSI)	*r* = 0.163 (−0.369/0.579)
	CO_av_	*r* = −0.312 (−0.684/0.208)
WCS_PL_ (AU)	CMJ	*r* = 0.258 (−0.657/0.254)
	Log_10_ (mRSI)	*r* = −0.009 (−0.488/0.474)
	CO_av_	*r* = −0.519* (−0.794/−0.035)

Abbreviations: ACC_>4_ = accelerations above 4 m/s^2^, ACC_max_ = maximum acceleration, CI = confidence interval, CMJ = countermovement jump, CO_av_ = average distance in the modified single‐leg triple crossover hop test, DEC_>−4_ = decelerations above −4 m/s^2^, DEC_max_ = maximum deceleration, HSR_>21_ = high‐speed running distance above 21 km/h, m/min = meterage per minute, PL/min = player load per minute, mRSI = modified reactive strength index, *V*_max_ = maximum velocity, WCS_dist_ = worst‐case scenario for distance, WCS_PL_ = worst‐case scenario for player load.

^*^ *p* < 0.05.

^**^ *p* < 0.01.

#### 3.1.1 Countermovement Jump

A strong positive correlation was observed between CMJ jump height and ACC_max_ (*r* = 0.553, *p* < 0.05). Additionally, CMJ jump height and ACC_>4_ also showed a strong positive correlation (*r* = 0.655, *p* < 0.01). No significant correlations were found between CMJ and other physical match performance parameters (*p* >0.05).

#### 3.1.2 Modified Reactive Strength Index

A strong positive correlation was found between log_10_ (mRSI) and ACC_max_ (*r* = 0.513, *p* < 0.05). Additionally, log_10_ (mRSI) and ACC_>4_ also showed a strong positive correlation (*r* = 0.609, *p* < 0.01). No significant correlations were found between log_10_ (mRSI) and other physical match performance parameters (*p* >0.05).

#### 3.1.3 Single‐Leg Triple Crossover Hop Test

A strong negative correlation was found between CO_av_ and WCS_PL_ (*r* = −0.519, *p* < 0.05). No other physical performance parameters were significantly correlated with CO_av_ (*p* >0.05).

#### 3.1.4 Regressions

Statistical analysis provided valuable insights into the relationships between performance metrics and acceleration (Table 4).

**TABLE 4:** Equations of the linear regression models between physical match performance and strength tests.

Equation N°	Prediction model	*R* ^2^	Adjusted *R* ^2^	Cohen's *f* ^2^	95% CI	SEE	RSS
1**	${\text{ACC}}_{>4}=\left(0.005\times \text{CMJ}\right)-0.154$	42.9%	39.1%	0.750	−0.078 to 3.209	0.023	0.0076
2**	${\text{ACC}}_{>4}=\left(0.196\times \log_{10}\left(\text{mRSI}\right)\right)+0.230$	37.1%	32.9%	0.590	−0.133 to 2.732	0.024	0.0084
3*	${\text{ACC}}_{\max}=0.040\times \text{CMJ}+3.090$	30.6%	25.9%	0.440	−0.183 to 2.289	0.241	0.871
4*	${\text{ACC}}_{\max}=\left(1.599\times \log_{10}\left(\text{mRSI}\right)\right)+4.542$	26.3%	21.4%	0.357	−0.211 to 2.043	0.248	0.925
5*	${\text{WCS}}_{\text{PL}}=\left(-9.804\times {\text{CO}}_{\text{av}}\right)+122.766$	27.0%	22.1%	0.370	−0.207 to 2.079	5.983	536.9

Abbreviations: ACC_>4_ = accelerations above 4 m/s^2^, ACC_max_ = maximum acceleration, CI = confidence interval, CMJ = countermovement jump, CO_av_ = average distance in the modified single‐leg triple crossover hop test, SEE = standard error estimate, RSS = residual sum of squares, WCS_PL_ = worst‐case scenario for player load.

^*^ *p* < 0.05.

^**^ *p* < 0.01.

**FIGURE 4:**
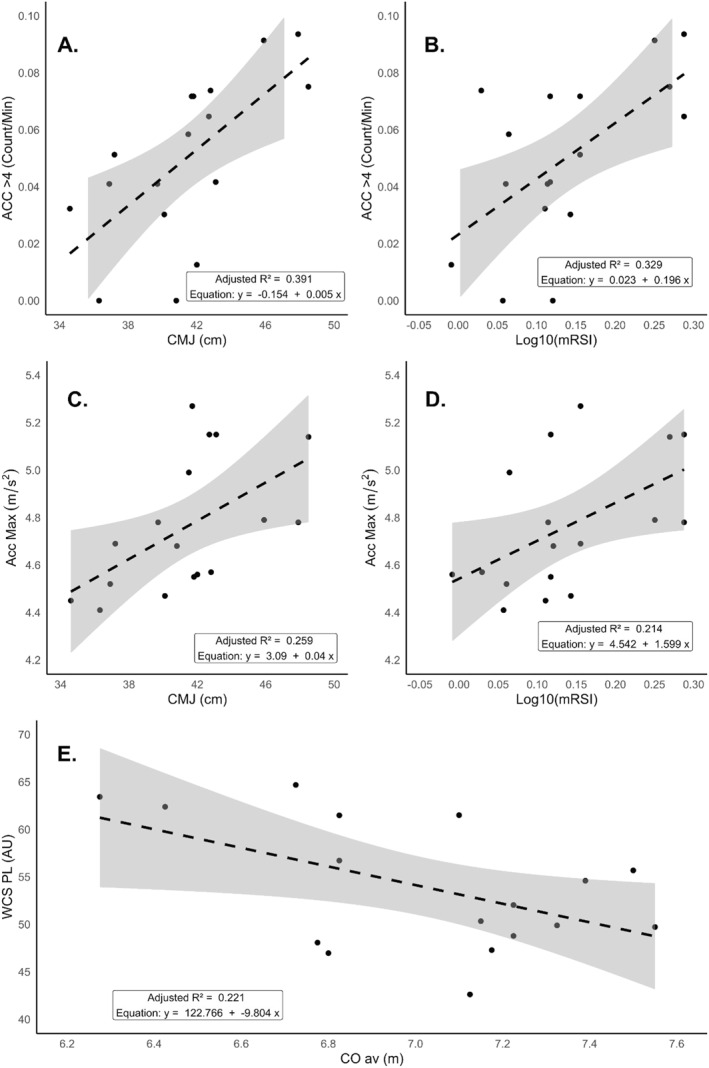
Linear regression models (±95% CI) of (A) CMJ and ACC_>4_, (B) log_10_ (mRSI) and ACC_>4_, (C) CMJ and ACC_max_, (D) log_10_ (mRSI) and ACC_max_ and (E) CO_av_ and WCS_PL_ inclusive of prediction equation and adjusted *R* ^2^.

For ACC_>4_, two separate prediction models were found to be statistically significant. Equation 1 shows that CMJ height significantly predicted ACC_>4_ (*F*(1,15) = 11.257, *p* < 0.01). In addition to CMJ, mRSI significantly predicted ACC_>4_, as shown in Equation 2 (*F*(1,15) = 8.844, *p* < 0.01). Figure 4A,B illustrates these relationships, respectively.

For ACC_max_, two significant models emerged, demonstrating that both CMJ height and log_10_ (mRSI) significantly predicted ACC_max_. Equation 3 demonstrates that CMJ height significantly predicted ACC_max_ (*F*(1,15) = 6.603, *p* < 0.05). Equation 4 reveals that log_10_ (mRSI) significantly predicted ACC_max_ (*F*(1,15) = 5.362, *p* < 0.05). Figure 4C,D corresponds to Equations 3 and 4, respectively, with CMJ and log_10_ (mRSI) predicting ACC_max_.

In addition to the acceleration models, Equation 5 (Figure 4E) shows that CO_av_ significantly predicted the WCS_PL_ (*F*(1,15) = 5.543, *p* < 0.05), although it had a smaller effect size than the models predicting acceleration.

## 4 Discussion

The main aim of this study was to examine associations between different strength capacities, fast MDS and reactive strength, and GPS‐derived physical match performance. Partially confirming our hypothesis, we found significant correlations and predictive relationships between fast MDS (via CMJ), reactive strength (via mRSI) and key acceleration metrics (ACC_max_ and ACC_>4_). Additionally, CO_av_ was a significant predictor of WCS_PL_. Regarding our secondary aim, although regression models explained under 40% of variance, this aligns with existing literature in sports performance, where predictive power typically ranges from 20% to 50% due to the complex nature of match play involving technical, tactical, psychological and contextual factors (Sarmento et al. 2018; Bergkamp et al. 2019). For instance, Oliva‐Lozano et al. (2024) reported 100% variance explained in a composite performance index using PLS‐SEM, but this relied on multiple latent constructs across diverse indicators.

In contrast, our models identified meaningful variance using only a few key neuromuscular predictors, underscoring the relevance of fast MDS and reactive strength in supporting high‐intensity accelerations. These findings reinforce the utility of strength diagnostics in athlete monitoring and training design. Fast MDS, measured by CMJ, showed a strong positive correlation with ACC_max_ and a moderate positive correlation with higher acceleration capacity and frequency of intense efforts. Similarly, reactive strength (log_10_ (mRSI)) correlated strongly positively with both ACC_max_ and ACC_>4_, suggesting that athletes with higher reactive strength perform more frequent and intense accelerations during matches. Neither CMJ nor mRSI correlated significantly with other match performance variables. These findings highlight vertical fast MDS and reactive strength as specific contributors to acceleration‐related performance in soccer.

Regression analyses further explored these associations, with two models predicting ACC_>4_. When considering high‐intensity accelerations (ACC_>4_), CMJ height emerged as a significant predictor, with a large effect size (Cohen's *f* ^2^) and moderate explanatory power (*R* ^2^/adjusted *R* ^2^). Model diagnostics (SEE, RSS) also supported good model fit and predictive accuracy. Log_10_ (mRSI) also significantly predicted ACC_>4_, though with a smaller effect size and lower *R* ^2^ values. Despite this, the model still reflected a strong model fit for predicting acceleration based on the mRSI. Overall, fast MDS provided a stronger and more predictive model for ACC_>4_ than reactive strength, suggesting its greater relevance for high‐intensity acceleration demands in match play.

For ACC_max_, CMJ and log_10_ (mRSI) both predicted maximum acceleration capacity, as hypothesised. When examining the players' maximum acceleration, CMJ (Equation 3) predicted maximum acceleration with a medium effect size. The *R* ^2^ and adjusted *R* ^2^ values explained a smaller proportion of the variance compared to previous models, indicating a weaker, though still meaningful, fit. In contrast, reactive strength (Equation 4) also showed a medium effect size. However, lower *R* ^2^ values and higher SEE/RSS indicated a relatively weaker fit than Equation 3. Thus, vertical fast MDS appears to be a stronger predictor of ACC_max_ than reactive strength.

Finally, regarding CO_av_, a strong negative correlation was found with the WCS_PL_, indicating that shorter CO_av_ distances were associated with a greater external workload during the WCS_PL_. No other significant correlations were observed between CO_av_ and physical performance parameters. These findings suggest that athletes with greater horizontal fast MDS are better able to meet the WCS_PL_ demands and accumulate less PL. This underscores the practical relevance of horizontal force production during high‐intensity periods such as WCS_PL_. Coaches and practitioners may consider using CO_av_ as a performance monitoring metric to identify limitations in an athlete's horizontal force application.

Targeted neuromuscular training, such as resisted sprints (Morin et al. 2017), horizontal plyometrics (Jiménez‐Reyes et al. 2017) or conical pulley exercises (Gonzalo‐Skok et al. 2022), may improve this mechanical profile. Additionally, tracking CO_av_ across the season may help adjust training loads and optimise recovery, especially during congested fixture periods where managing fatigue is critical. As hypothesised, regression analysis revealed that CO_av_ significantly predicted WCS_PL_, with a negative impact. Only horizontal fast MDS, measured via CO_av_, was able to predict WCS_PL_, with a medium effect size. The model's *R* ^2^ and adjusted *R* ^2^ values showed moderate explanatory power, whereas SEE and RSS indicated that it accounted for a meaningful portion of the variance.

Overall, the models varied in explanatory power and effect sizes, with ACC_>4_ showing the strongest relationships with strength measures. The effect sizes (Cohen's *f* ^2^) ranged from medium to large, highlighting the predictive value of both CMJ and mRSI for acceleration outcomes. Practically, the large effect size for vertical fast MDS (CMJ height) suggests that even modest gains can lead to meaningful improvements in high‐intensity acceleration performance during match play. This aligns with recent evidence showing that plyometric and sprint training enhance CMJ and short‐distance acceleration in team sport athletes (Zheng et al. 2025). Similarly, the moderate effect size for reactive strength (log_10_ (mRSI)) suggests that players with more efficient stretch‐shortening cycle function can achieve higher acceleration outputs, however with slightly lower predictive strength than CMJ.

This supports findings that RSI‐based reactive strength is associated with rapid force production and sprint performance in elite athletes (Jarvis et al. 2022). Together, these insights reinforce the value of vertical reactive strength development, via plyometrics, speed‐strength drills and reactive jump training, in soccer conditioning programmes.

### 4.1 Underlying Mechanisms

The observed associations between strength capacities and match performance likely reflect underlying neuromechanical mechanisms. Vertical strength, assessed via countermovement jump (CMJ) performance, reflects an athlete's capacity to generate rapid concentric force (Cormie et al. 2011), critical for vertical displacement and explosive actions such as jumping and sudden directional changes (Suchomel et al. 2016). Additionally, the CMJ mirrors the biomechanical pattern used in acceleration, specifically, the triple extension of the ankle, knee and hip, enhancing its functional relevance to sprinting performance (Loturco et al. 2019). Reactive strength, measured through mRSI, reflects the efficiency of the stretch‐shortening cycle, enabling rapid transitions between eccentric and concentric muscle actions, which support acceleration actions during gameplay (Jarvis et al. 2022).

Horizontal strength, evaluated via CO_av_, captures the ability to generate force in the horizontal plane, a key factor in sprint acceleration and high‐intensity running (Maulder and Cronin 2005). Efficient horizontal force application reduces the mechanical cost of acceleration, potentially explaining the inverse relationship between horizontal strength and accumulated workload during matches. These findings align with recent research emphasising the role of force orientation and neuromuscular efficiency in sprint performance and workload management in team sports.

### 4.2 Accelerations and Decelerations

The scientific literature presents conflicting findings regarding the relationship between lower‐body strength capacities and acceleration performance. Consistent with our findings, Rago (Rago et al. 2018) reported associations between match‐based acceleration parameters and CMJ performance in male athletes. However, methodological differences should be noted, as Rago's study assessed total distance covered while accelerating above 3 m/s^2^, whereas our study counted the number of accelerations exceeding a higher threshold (>4 m/s^2^). This distinction is important, as recent research has shown that using a 3‐m/s^2^ threshold may overestimate true acceleration loads (Nevado Garrosa et al. 2025); a higher cut‐off could better capture high‐intensity actions relevant to match demands. They also reported associations with deceleration parameters, which may be due to differences in measurement methods and thresholds (>–3 m/s^2^) compared to our study. Again, these differences could be attributed to the measurement (distance covered vs. count) and the threshold applied (>−3 m/s^2^).

In contrast to our findings, Rebelo (Rebelo et al. 2016) reported unclear associations between acceleration counts above 3 m/s^2^ during small‐sided games (SSGs) and CMJ performance. Similarly, deceleration measures in SSGs also showed no clear association with CMJ. This may be due to the distinct biomechanical demands of SSGs, which involve smaller playing areas, more frequent directional changes and lower peak running velocities compared to full‐match scenarios (Lacome et al. 2018). Finally, contrary to our results, Pedersen (Pedersen et al. 2022) found no association between accelerations and CMJ performance. This discrepancy may be due to differences in study design, including a female sample and the use of lower acceleration thresholds (>±2 m/s^2^), which may not adequately reflect high‐intensity demands (Randell et al. 2021).

### 4.3 Total Distance

Most of the scientific literature aligns with our findings, indicating that fast MDS performance is not associated with TD covered during a match (Pedersen et al. 2022; Buchheit et al. 2010; Rampinini et al. 2007). In contrast, Rebelo (Rebelo et al. 2016) reported an association between CMJ performance and TD covered during SSG, but this relationship was only evident in the smaller version of the SSG. Larger SSGs, which more closely resemble match conditions, observed findings consistent with our study, showing no significant association between TD and CMJ. Similarly, one study reported a moderate correlation between CMJ and TD during friendly matches (Rago et al. 2018). Notably, the authors did not offer a clear explanation for this discrepancy, despite its inconsistency with other published findings. This lack of association may emerge from the fact that TD reflects contextual and tactical elements, such as team strategy and match dynamics, rather than intrinsic physical capacities such as strength (Mandorino et al. 2025).

### 4.4 Velocity

The scientific literature also presents mixed findings regarding the relationship between lower‐body strength and velocity‐based performance measures. In our study, no associations were found between any strength capacities and velocity‐related parameters, such as HSR_>21_ or *V*_max_. Consistent with our results, several studies have also reported no associations between HSR and strength capacities (Altmann et al. 2018; Silva et al. 2013; Pedersen et al. 2022; Rampinini et al. 2007). Likewise, two studies found no significant relationship between *V*_max_ and CMJ (Rago et al. 2018; Pedersen et al. 2022). Only Rebelo (Rebelo et al. 2016) reported a moderate association between HSR and strength during SSGs, though this was limited to the smaller formats. Larger SSGs, more comparable to real match conditions, showed no such association, aligning with our findings. This may be explained by the spatial constraints of smaller SSGs, where players rely more heavily on acceleration to reach high speeds.

In contrast, larger formats provide more space and time to build velocity, potentially reducing the direct influence of strength on reaching HSR. As previously observed in the literature, vertical jump and linear sprinting are generally regarded as independent skills (Gonzalo‐Skok et al. 2022). Discrepancies in other studies may be due to the use of lower HSR thresholds, different age groups or measured *V*_max_ in isolation (Rago et al. 2018; Jarvis et al. 2022; Buchheit et al. 2010; Wheeler et al. 2023). Although no studies directly link reactive strength to soccer performance, prior research has shown that greater lower‐leg stiffness is associated with improved running and linear sprint speeds (Maloney and Fletcher 2021). However, our results do not support this relationship, possibly due to the limited distances required to reach maximum velocity during match play and the relatively short time spent at *V*_max_ (Bangsbo et al. 2006; Weakley et al. 2023). Furthermore, a recent review evaluated GPS accuracy and questioned its reliability for measuring *V*_max_ during soccer matches, suggesting caution in interpreting this parameter (Zabaloy et al. 2024).

### 4.5 Worst‐Case Scenario

A limited body of research has investigated the relationship between WCS and strength capacities. Consistent with our findings, Silva (Silva et al. 2013) reported no associations between WCS_dist_ and CMJ performance, a measure of fast MDS in the vertical plane. In contrast, our study found a significant association between WCS_PL_ and CO_av_, a measure of horizontal fast MDS. This relationship suggests that higher horizontal strength capacities may help mitigate the external workload experienced during WCS_PL_ periods. In other words, players with superior horizontal fast MDS may require fewer high‐intensity efforts to meet the demands of the WCS. This could have a positive effect on injury prevention, as the players are exposed to lower levels of external load during the most demanding scenarios by reducing the exposure to repeated high‐intensity actions. Overall, these findings contribute to the understanding of the relationship between WCS and strength capacities, highlighting the potential benefits of specific strength capacities in mitigating fatigue, improving performance and potentially decreasing injury risk during high‐load match conditions.

### 4.6 Limitations

The primary limitation of this study was its sample size. As indicated by the power analysis, a larger sample would be needed to achieve 80% power for detecting medium and large effect sizes. For vertical strength assessment, the jump height was determined using the flight time method, which is subject to small measurement errors. The use of force platforms could improve accuracy by calculating jump height via the take‐off velocity method and distinguishing between jump phases, offering more detailed insights into movement execution (Comfort et al. 2018). Regarding mRSI, some athletes may have been better suited to a higher box height, which could more effectively capture their use of the rapid stretch‐shortening cycle. Finally, GPS data lacked context on match events; incorporating video analysis could clarify the actions linked to these measurements.

### 4.7 Future Directions

Future research should investigate additional strength capacities beyond fast MDS and reactive strength, such as heavy MDS, maximum isometric strength, explosive strength and eccentric strength. Eccentric strength may play a critical role in deceleration, change‐of‐direction ability and injury prevention, yet its relationship with match‐based performance indicators remains underexplored.

Studies could incorporate isokinetic or flywheel‐based assessments to better evaluate eccentric strength and its relationship to high‐intensity match actions. Likewise, integrating measures of maximal isometric and explosive strength could offer a more comprehensive profile of neuromuscular function and its impact on performance. Clarifying how different strength capacities relate to high‐intensity actions during match play would deepen our understanding of neuromuscular performance demands. Additionally, synchronising GPS data with video footage would enhance contextual analysis, helping to identify specific actions linked to GPS outputs and the physical qualities that drive them. This multifaceted approach could enhance our understanding of how specific strength qualities contribute to key performance outcomes in football.

### 4.8 Conclusions

Fast MDS and reactive strength were positively associated with maximum in‐game acceleration capacity and the number of high‐intensity accelerations. Lower horizontal fast MDS was linked to higher workloads during the most intense game periods (WCS_PL_). These associations highlight specific strength qualities as key indicators of match performance, which coaches can target to optimise training and monitoring strategies.

## Author Contributions

J.R.L. contributed to the conceptualization and design of the study, data curation, formal analysis, investigation, methodology, project administration, resources, software, visualization, and writing of the original draft. V.P.H. contributed to data curation, investigation, methodology, supervision, validation, and writing – review and editing. J.S.S. contributed to conceptualization, data curation, formal analysis, investigation, methodology, supervision, and writing – review and editing. C.B.F. contributed to formal analysis, project administration, supervision, visualization, and writing – review and editing. All authors contributed to the revision of the manuscript, read, and approved the final version for publication.

## Conflicts of Interest

The authors declare no conflicts of interest.
